# Co-treatment with grape seed extract and mesenchymal stem cells in vivo regenerated beta cells of islets of Langerhans in pancreas of type I-induced diabetic rats

**DOI:** 10.1186/s13287-022-03218-y

**Published:** 2022-12-21

**Authors:** Alyaa Farid, Hebatallah Haridyy, Salma Ashraf, Selim Ahmed, Gehan Safwat

**Affiliations:** 1 Zoology Department, Faculty of Science, Cairo University, Giza, Egypt; 2 Biotechnology Department, Faculty of Science, Cairo University, Giza, Egypt; 3 Faculty of Biotechnology, October University for Modern Sciences and Arts (MSA), Giza, Egypt

**Keywords:** Diabetes mellitus, GSE, MSC, NF-κB, Glucose, Insulin

## Abstract

**Background:**

Nowadays, diabetes mellitus is known as a silent killer because individual is not aware that he has the disease till the development of its complications. Many researchers have studied the use of stem cells in treatment of both types of diabetes. Mesenchymal stem cells (MSCs) hold a lot of potential for regenerative therapy. MSCs migrate and home at the damaged site, where they can aid in the repair of damaged tissues and restoring their function. Oxidative stress and inflammation represent a huge obstacle during MSCs transplantation. Therefore, the present study aimed to evaluate the role of grape seed extract (GSE) administration during MSCs transplantation in streptozotocin (STZ)-induced type I diabetes. Furthermore, testing some of GSE components [procyanidins(P)-B1 and P-C1] in conjunction with MSCs, in vivo, was performed to determine if one of them was more effective in relieving the measured attributes of diabetes more than the whole GSE.

**Methods:**

Firstly, GSE was prepared from the seeds of Muscat of Alexandria grapes and characterized to identify its phytochemical components. Experimental design was composed of control group I, untreated diabetic group II, GSE (300 mg/kg)-treated diabetic group III, MSCs (2 × 10^6^ cells/rat)-treated diabetic group IV and GSE (300 mg/kg)/MSCs (2 × 10^6^ cells/rat)-treated diabetic group V. Type I diabetes was induced in rats by intravenous injection with 65 mg/kg of STZ. Treatment started when fasting blood glucose (FBG) level was more than 200 mg/dl; GSE oral administration started in the same day after MSCs intravenous injection and continued daily for 30 consecutive days.

**Results:**

The results showed that GSE/MSCs therapy in type I-induced diabetic rats has dramatically managed homeostasis of glucose and insulin secretion; together with, improvement in levels of inflammatory markers and oxidative stress.

**Conclusion:**

Co-treatment with GSE and MSCs in vivo regenerates beta cells in type I-induced diabetic rats.

## Introduction

Diabetes mellitus is a long-term metabolic syndrome caused by the lack of and/or resistance to insulin. Cardiovascular disease, retinopathy, nephropathy, angiopathy, and neuropathy are all complications induced by the excessive glucose levels and protein glycation. Type I diabetes is developed in children due to the autoimmune attack of beta cells that secret insulin leading to its deficiency and hyperglycemia; therefore, it is mainly treated with insulin [1]. It affects about half percent of the general population and is growing at a rate of 3% each year, causing serious concern among healthcare professionals all over the world. Microbial infection, environmental pollution, and the seasons of winter and autumn are all known to trigger type I diabetes [2].

Common insulin therapies, over time, become ineffective in controlling high blood glucose levels. Islet transplantation therapy is limited due to a scarcity of pancreatic cells, rejection of transplanted cells and the usage of immunosuppressive drugs [3]. Mesenchymal stem cells (MSCs) therapies may be able to help overcome these limitations due to their low immunogenic potential, immunological privilege, and immunomodulatory properties [4]. MSCs can differentiate into a variety of cell types, which can then be used to replace damaged tissues and restore their function after local engraftment [5]. However, a lack of standardized protocols, the difficulty of in vivo differentiation, and the risk of tumor formation limit MSCs' potential for stem cell treatment [6]. Campisi and d'Adda di Fagagna [7] reported that MSCs may lose their activity and enter senescence due to intrinsic and/or external factors like DNA damage, stimulation of cytokine secretion and widespread tissue damages. Senescence has two faces as it shields cells from injury and prevents activation of oncogene; and on the other hand, it inhibits tissue regeneration leading to age-related degeneration [8]. Jeong and Cho [9] reported that although reactive oxygen species released from MSCs maintain their proliferation and/or differentiation, it causes DNA damages and impairs MSCs function.

For medical professionals, the most important problem is how to treat diabetes mellitus without creating any negative effects. Eight hundred medicinal plants have been used to prevent diabetes, according to the World Ethanobotanical Organization. In recent years, grape seed extract (GSE) has become increasingly popular as a nutritional supplement in many countries [10]. This is due to the fact that grape seeds are high in phenolic chemicals, which may have many health benefits [11]. GSE is able to scavenge superoxide radicals due to its anti-oxidant properties [12]. Flavan-3-ol, which includes proanthocyanidins and catechins, is abundant in GSE [13]. GSE has significant levels of polyphenol proanthocyanidins like procyanidins, prodelphinidins and propelargonidins, which are oligomers of flavan-3-ol molecules like catechin and epicatechin [14]. Proanthocyanidins have been found to have significant antioxidant activity and scavenge reactive oxygen and nitrogen species in vitro, as well as regulate immunological function and platelet activation, and induce vasorelaxation effect by increasing the nitric oxide (NO) release from endothelium [15]. Furthermore, proanthocyanidins slow the advancement of atherosclerosis and reduce the accumulation of low-density lipoprotein (LDL) cholesterol [16].

Therefore, the present study aims to evaluate the effect of GSE during MSCs transplantation in type I-induced diabetic rats. The possibility that GSE can aid in decreasing inflammation and oxidative stress, which result from diabetes induction and MSCs expansion, was examined. This was accomplished through biochemical, immunological and histopathological evaluation. Furthermore, testing some of GSE components [procyanidins(P)-B1 and P-C1] in conjunction with MSCs, in vivo, was performed to determine if one of them was more effective in relieving the measured attributes of diabetes more than the whole GSE. In this study, we purified and characterized the seeds of Muscat of Alexandria grapes in our laboratory to provide a simple protocol for preparation of GSE. However, many companies provide grape seeds proanthocyanidins extract like Sigma-Aldrich (222838-60-0).

## Materials and methods

### Isolation and characterization of bone marrow mesenchymal stem cells (BM-MSCs)

The femora of ten rats, anesthetized with intraperitoneal injection with 30 mg/kg of sodium pentobarbital, were aseptically harvested using phosphate-buffered saline. The collected cells were suspended (at 10^6^ cells/ml) in RPMI-1640 medium containing glutamine, 5% fetal bovine serum, gentamicin–amphotericin B (30 µg/ml gentamicin and 15 ng/ml amphotericin-B) and fibroblast growth factor (8 ng/ml). MSCs culture was supplemented with fetal bovine serum (10%) and penicillin–streptomycin (1%, 100 IU/ml penicillin and 100 μg/mL) and incubated in 5% CO_2_ at 37 °C until reaching 80 to 90% confluence. After fourteen days, trypsin (0.25%) was used to detach the cells followed by washing with phosphate-buffered saline several times. Passage three of MSCs was used for the study [17]. Flow cytometry was used to analyze cell surface markers CD 34, 45, 90 and 105. PKH26 fluorescent linker dye (PKH26GL, Sigma-Aldrich, St. Louis, USA) was used to label MSCs according to Elberry et al*.* [18]. Briefly, cells were suspended in Diluent C (one ml), followed by mixing with an equal volume of the PKH26 solution (4 nM) and incubation for five minutes at 25 °C.

### Preparation of GSE

Seeds of Muscat of Alexandria grapes were isolated and dried at room temperature in the shade before being crushed into a fine powder. According to Mandic et al*.* [19], 100 g of grape seeds powder was blended with 40 ml H_2_O and 360 ml ethyl alcohol. The alcohol was evaporated below 40 °C under reduced pressure after the GSE was filtered with filter paper.

### Chemical composition of grape seeds

Characterization of grape seeds was performed by the microanalytical center, Cairo University. Where the moisture, ash, fibers content, total lipid, protein and carbohydrates were determined according to the official methods of the association of official analytical chemist [20].

### Characterization of GSE

Using the Folin–Ciocalteau process, the sum of total soluble polyphenols in the extracts was calculated spectrophotometrically [21]. The findings were expressed as gallic acid equivalents in grams/ 1 kg of dry seed weight using gallic acid as a calibration standard. Using (+)-catechin as a standard, the sum of total flavan-3-ols was determined colorimetrically using the vanillin process [22, 23]. Catechin equivalents in grams/1 kg dry seed weight were used to measure the results. All of the tests were performed three times, and the results were averaged. GSE was analyzed by HPLC for the determination of the distribution percent (%) of phenolic compounds.

### Screening of phytochemicals in GSE

The phytoconstituents in grape seeds were determined according to the methods of Djeridane et al*.* [24]. Total polyphenols were measured using the Folin–Ciocalteu method, according to Maurya and Singh [25] and its value was expressed as milligram gallic acid/gram of dry seed. The flavonoid content of GSE was measured according to the methods of Ebrahimzadeh et al*.* [26]. Flavonoids content was expressed as milligrams quercetin/gram of dry seed.

### Analysis of the total antioxidant capacity of GSE

The antioxidant activities of the GSE were evaluated as the measure of radical scavenging using 2,2-diphenyl-1-picryl-hydrazyl-hydrate (DPPH) according to Brand-Williams et al*.* [27]. The radical scavenging (%) = [((A0 − A1)/A0) × 100]; where: A0 and A1 were the absorbance of control and sample extracts, respectively. The 50% inhibitory concentration value (IC50) was indicated as the effective concentration of the sample that was required to scavenge 50% of the DPPH free radicals.

### Induction of type I diabetes

Sprague Dawley male rats (7 weeks old; weighting; 180–220 g) were purchased from TBRI. Rats were intravenously injected with 65 mg/kg of streptozotocin (STZ, Sigma, MA, USA) [32.5 mg of STZ dissolved in 50 mM sodium citrate buffer (pH: 4.5, Fisher)] according to Furman [28]. Animals were fasted for six to eight hours before STZ administration. On the 1st day after injection, rats were provided with normal diet and 10% sucrose solution. Rats with fasting blood glucose (FBG) level˃ 200 mg/dl were considered diabetic.

### Experimental design

Forty Sprague Dawley male rats were randomly divided into five groups (*n* = 8); gp I: healthy control, gp II: untreated induced type I diabetic group, gp III: GSE-treated diabetic group, gp IV: MSCs-treated diabetic group and gp V: GSE- and MSCs-treated diabetic group. Random numbers were generated using the standard = RAND() function in Microsoft Excel. The researchers were blinded to treatment allocation throughout the entire duration of the study. Rats received (2 × 10^6^ cells/rat) MSCs in 0.2 ml Dulbecco’s modified Eagles medium (DMEM) by intravenous injection. GSE was dissolved in distilled water and orally administrated at 8.00 am, after MSCs injection, in a daily dose of 300 mg/kg for 30 consecutive days [29]. Normal diet and water were provided ad libitum; animals were maintained one week before the experiment for acclimatization. Animals were observed twice a day by the researchers. Weight and FBG were checked, as well as the intake of food and water and a general evaluation of the animal's activity, panting, and fur appearance. The protocol, all experimental procedure and animal maintenance were approved by the Institutional Animal Care and Use Committee, Cairo University, Egypt. Sample size was calculated, based on the ethics committee instructions, by the aid of http://www.biomath.info/power/prt.htm. The primary outcome of the study was decreasing FBG level, and based on power calculation, 8 rats/group were needed. The manuscript reporting was adhered to the ARRIVE guidelines for the reporting of animal experiments. At the end of experiments (30 days), blood samples were collected from all experimental groups for serum preparation, in addition to pancreatic, liver and kidney samples for histological and immunohistochemicals studies.

### Evaluation of GSE procyanidins in MSCs protection in vivo

Procyanidin (P)-B1 (20315-25-7) and P-C1 (37064-30-5) were purchased from Sigma-Aldrich, MA, USA. Twenty-five Sprague Dawley male rats were divided into five groups (*n* = 5); gp I: healthy control, gp II: untreated induced type I diabetic group, gp III: P-B1/MSCs-treated diabetic group, gp IV: P-C1/MSCs-treated diabetic group and gp V: GSE/MSCs-treated diabetic group. Induction of diabetes and protocol of MSCs transplantation were performed as previously described. P-B1, P-C1 or whole GSE were dissolved in distilled water and orally administrated, after MSCs injection, in a daily dose of 300 mg/kg for 30 consecutive days. At the end of experiments (30 days), blood samples were collected from all experimental groups for serum preparation, in addition to pancreatic samples for determination of MSCs homing.

### Biochemical parameters in serum

Fructosamine (FTA) level was measured, by ab228558 fructosamine assay kit (Abcam, MA, USA), to detect the average glycemic level over the past three weeks [30, 31]. Fasting blood glucose (FBG) and serum insulin were measured using ab65333 glucose assay kit (Abcam, MA, USA), and insulin ELISA kit (CSB-E05070r, CUSABIO, Texas, USA). Serum liver function enzymes [alanine aminotransferase (ALT) and aspartate aminotransferase (AST)] were measured by rat ELISA kits (MBS269614, San Diego, California, USA and CSB-E13023r, CUSABIO, Texas, USA; respectively). Serum urea and creatinine were measured by ELISA kits (MBS2600001, San Diego, California, USA and MBS007289, San Diego, California, USA; respectively). Serum malondialdehyde (MDA), superoxide dismutase (SOD) and glutathione peroxidase (GPx) were measured by rat ELISA kits (MBS268427, San Diego, California, USA; MBS266897, San Diego, California, USA and MBS727547, San Diego, California, USA), respectively.

### Preparation of pancreatic homogenate

One gram of dissected pancreatic tissue was homogenized in 4.5 ml of cold Tris–HCl buffer (10 mmol, pH = 7.4), followed by ten minutes centrifugation at 4 °C and 3000 rpm. Standard Lowry’s protocol [32] was used for measuring protein content in supernatant.

### Pancreatic oxidative stress and inflammation

Pancreatic levels of MDA and glutathione (GSH) were measured by rat ELISA kits (MBS268427, San Diego, California, USA; and MBS265966, San Diego, California, USA), respectively. In addition, IL-1β, IL-12, TNF-α and TLR-4 were determined using rat ELISA kits (E-EL-R0012, elabscience, Texas, USA; CSB-E07364r, CUSABIO, Texas, USA; Biolegend 438,206, San Diego, California, USA and MBS705488, San Diego, California, USA), respectively, according to the manufacturer’s protocols.

### Histological and immunohistochemical studies

10% buffered formalin was used to fix organs from the different experimental groups, followed by dehydration, clearing with xylene and embedding in paraffin wax. Sections (4 μm) were stained with hematoxylin and eosin (H&E) and mounted. For immunohistochemical staining, paraffin pancreatic sections (4 μm) were deparaffinized in xylene, rehydrated in graded alcohols and washed by PBS. 3% hydrogen peroxide was used to block endogenous peroxidase activity followed by washing with PBS and blocking in 5% bovine serum albumin for 60 min. Pancreatic sections were incubated, for half an hour, with primary antibody [anti-nuclear factor-kappa beta (NF-кB) p65 antibody (phospho S536, ab86299, abcam, MA, USA) or monoclonal anti-insulin antibody (I2018, Sigma)] followed by washing with PBS. Sections were incubated, for 60 min, with secondary antibody [HRP conjugated rabbit anti-rat IgG, ab6734, abcam, MA, USA]. Chromogen (3, 3-diaminobenzidine, DAB) was used for color development, where brown color indicates positive result. Sections were washed and counterstained with 0.1% hematoxylin. Histological scoring of pancreas was performed according to Lutgendorff et al*.* [33]; 0 to 3 for each of necrosis, inflammatory cell infiltration and edema (Table 1).

**Table 1:** Histological scoring in pancreas

Parameter	Score	Indication
Necrosis	0 1 2 3	No necrosis Focal necrosis Partially necrotic/ulcerated Totally necrotic/ulcerated
Inflammatory cell infiltrate	0 1 2 3	No inflammatory cells Mild Moderate Marked
Edema	0 1 2 3	Absent Mild Moderate Marked

Morphometric analysis, for pancreatic sections, was performed to determine the degree of improvement in each group (Table 2) according to Farid et al*.* [31].

**Table 2:** Parameters for morphometric analysis of pancreas

Parameter	Score	Indication
Islet size reduction	0 1 2	Average Small Atrophied
Cellularity	0 1 2	Average Hypocelluar Acellular
Beta cell damage	0 1 2	Average Few/apoptotic Necrotic/absent
Capillaries dilation	0 1 2	Average Mildly dilated Markedly dilated/congested
Exocrine area abnormality	0 1 2	Average acini Small acini Atrophied acini
Ducts damage	0 1 2	Average Dilated Atrophied
Interstitium infiltration	0 1 2	Average Mildly dilated Markedly dilated/inflammatory infiltrate
Insulin secretion	0 1 2	Absent Mild Marked
NF-κB activity	0 1 2	Absent Mild Marked

Also, histopathological scoring of liver (Table 3) and kidney (Table 4) sections was performed according to Ansari et al*.* [34], Farid et al*.* [35] and Khalid et al*.* [36], respectively.

**Table 3:** Histological scoring in liver

Parameter	Score	Indication
Steatosis	0 1 2 3	Absent Mild Moderate Severe
Inflammatory cell infiltrate	0 1 2 3	No inflammatory cells Mild Moderate Marked
Capillaries dilation	0 1 2 3	Absent Mild Moderate Marked

**Table 4:** Histological scoring in kidney

Parameter	Score	Indication
Glomeruli size	0 1 2	Average Small size/congested Atrophied/necrotic
Bowman’s spaces dilation	0 1 2	absent Widened/dilated Obliterated
Tubules damage	0 1 2	Absent Moderate Severe
Inflammatory cell infiltrate	0 1 2	No inflammatory cells Moderate Marked
Capillaries dilation	0 1 2	Absent Moderate Marked

Examination was performed by an experienced blinded pathologist who is unaware of the experimental design; where, scoring used 20 random fields at × 400 magnification/animal (*n* = 8) for each experimental group.

### Statistical analysis

Data were evaluated with two-way ANOVA test and compared with post hoc test (Tukey test) [37]. The differences and similarities among the different experimental groups were examined by Duncan's multiple-range test. Results were expressed as mean ± SD. Values were considered significant at *p* < 0.05. For each group (*n* = 8), no animal was excluded from the analysis.

## Results

### Characterization of grape seeds

The chemical composition of grape seeds is represented in Table 5. Grape seeds contained 9.4% moisture, 2.6% ash, 28.2% carbohydrates, 35.5% fibers, 13.3% protein and 10.5% lipids. Moreover, total phenolic compounds and flavonoids in grape seeds were 11.6 mg gallic acid/g dry seed and 13.6 mg quercetin/g dry seed, respectively. The antioxidant activity of grape seeds was 89.2% with IC50 of 34.5 μg/ml. The average yield of GSE was 14.5 ± 1.1 g/kg dry grape seed. According to Folin–Ciocalteau method, the total polyphenols were 11.6 ± 2.1 g/kg dry grape seed. This content was approximately two times higher than the values obtained by HPLC method (5.1 ± 0.43 g/kg dry grape seed). The flavan-3-ols content in GSE was 11.1 ± 0.14 g/kg dry grape seed. The HPLC results are represented in Table 5. It was obvious that catechin (40.5 ± 1.21) and epicatechin (32.7 ± 0.81) were the predominant phenolic compound in GSE. The extract contained procyanidin B1, B2, B3, B4, B5 and C1, in addition to gallic acid and dimer gallate.

**Table 5:** Characterization and percent (%) of phenolic compounds in Muscat of Alexandria GSE

Item	Content
*Characterization of GSE*	
Moisture (%)	9.4 ± 2.4
Ash (%)	2.6 ± 0.7
Carbohydrates (%)	28.2 ± 1.6
Fibers (%)	35.5 ± 3.4
Protein (%)	13.3 ± 1.2
Total lipids (%)	10.5 ± 1.3
Total phenolic compounds (mg gallic acid/ g dry seed)	11.6 ± 2.1
Flavonoids (mg quercetin/g dry seed)	13.6 ± 3.4
Total antioxidant activity (DPPH; %)	89.2 ± 1.3
IC50(μg/ml)	34.5 ± 2.9
*Phenolic compounds (%)*	
( ±)-Catechin	40.5 ± 1.21
( −)-Epicatechin	32.7 ± 0.81
Procyanidin-B1	2.0 ± 0.42
Procyanidin-B2	3.4 ± 0.89
Procyanidin-B3	1.2 ± 2.02
Procyanidin-B4	4.1 ± 1.21
Procyanidin-B5	1.4 ± 0.43
Procyanidin-C1	3.6 ± 0.75
Gallic acid	5.6 ± 0.14
Dimer gallate	4.6 ± 0.77

### Characterization of MSCs

**Fig. 1:**
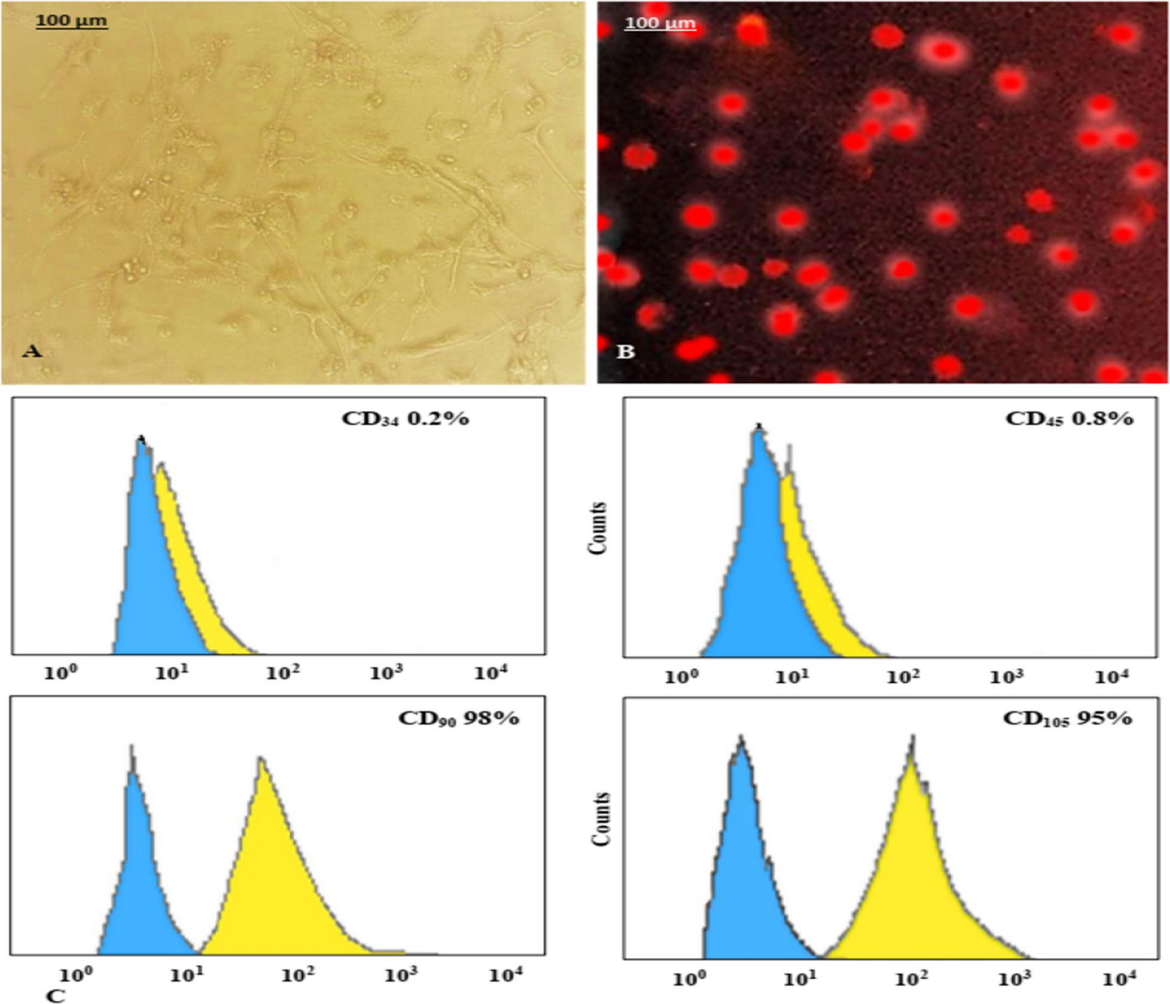
Cultured BM-MSCs photomicrograph (**A**, × 200), *ex vivo* BM-MSCs labeled with PKH26 (**B**, × 200) and flow cytometry results (**C**)

**Fig. 2:**
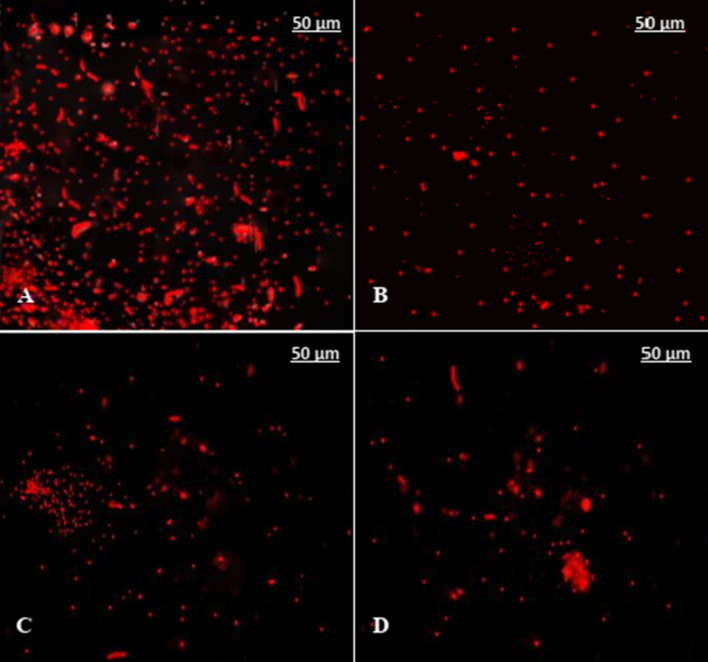
In vivo tracking of BM-MSCs in pancreas of Sprague Dawley male rats treated with GSE/MSCs (**A**), MSCs only (**B**), P-B1/MSCs (**C**) and P-C1/MSCs (**D**). MSCs were labeled with PKH26, × 200

After fourteen days of in vitro culture, MSCs have fibroblast-like morphology and were adhered to the flask surface (Fig. 1A); also, MSCs were ex vivo labeled with PKH26 and revealed high labeling efficiency (Fig. 1B). Flow cytometry analysis showed a negative staining for CD34 and CD45 markers and high expression of CD90 and CD105, indicating that the cultured cells were of mesenchymal origin as well as of high purity without hematopoietic lineage cell contamination (Fig. 1C). According to Fig. 2A, animals treated with GSE/MSCS showed high density of PKH26-labeled MSCs homing in pancreas more than those treated with MSCs only (Fig. 2B), P-B1/MSCs (Fig. 2C) or P-C1/MSCs (Fig. 2D).

### Effect of GSE and/or MSCs transplantation on body weight, FBG, serum insulin and FTA

Reduction in animals’ body weight was noticed upon diabetes induction (group II). Treatment in diabetic groups either by GSE alone or MSCs alone significantly improved the body weight (210.6 and 221.3 g for gp III and IV, respectively), but rats of group V showed normal body weight (255.7 g) when compared to control group I (250.3 g). Diabetes induction in group II elevated both of FBG (244.3 mg/dl) and FTA levels (299.4 μmol/mL) and decreased serum insulin level (5.2 μU/mL) when compared to negative control group I (Table 6). Administration of GSE with MSCs transplantation, in group V, significantly decreased FBG and FTA levels (95.4 and 159.6, respectively) and elevated serum insulin level (7.5) when compared to untreated diabetic group II, GSE-treated diabetic group III and MSCs-treated diabetic group IV (FBG/ gp II: 244.3, gp III: 135.4, gp IV: 121.4; FTA/ gp II: 299.4, gp III: 178.5, gp IV: 181.3; insulin/ gp II: 5.2, gp III: 5.9, gp IV: 6.1).

**Table 6:** Effect of GSE and/or MSCs transplantation on serum biochemical parameters in different experimental groups

Parameters	Healthy control group (I)	Untreated diabetic control group (II)	GSE-treated diabetic group (III)	MSCs-treated diabetic group (IV)	GSE/MSCs-treated diabetic group (V)
Body weight (gm)	250.3 ± 0.7^d^	180.3 ± 2.3^a^	210.6 ± 1.4^b^	221.3 ± 2.1^c^	255.7 ± 0.9^d^
FBG level (mg/dl)	97.2 ± 2.1^a^	244.3 ± 3.3^c^	135.4 ± 3.2^c^	121.4 ± 3.6^b^	95.4 ± 1.6^a^
Insulin (μU/mL)	7.2 ± 1.7c	5.2 ± 0.1^a^	5.9 ± 2.0^a^	6.1 ± 0.9^b^	7.5 ± 2.9^c^
FTA (μmol/L)	161.1 ± 0.4^a^	299.4 ± 4.9^c^	178.5 ± 2.5^b^	181.3 ± 2.1^b^	159.6 ± 4.1^a^
ALT (U/L)	30.2 ± 6.3^a^	60.1 ± 3.8^c^	42.5 ± 1.1^b^	39.1 ± 2.1^b^	29.4 ± 2.3^a^
AST (U/L)	25.4 ± 0.5^a^	40.1 ± 1.1^c^	30.1 ± 0.8^b^	29.4 ± 0.6^b^	23.1 ± 0.7^a^
Urea (mg/dl)	32.1 ± 3.1^a^	87.9 ± 2.1^c^	39.1 ± 3.6^b^	40.2 ± 1.2^b^	33.2 ± 0.3^a^
Creatinine (mg/dl)	0.4 ± 0.1^a^	1.3 ± 0.1^c^	0.5 ± 0.1^a^	0.6 ± 0.1^b^	0.4 ± 0.2^a^
Triglycerides mg/dl)	80.1 ± 0.9^a^	128.3 ± 6.2^c^	89.4 ± 4.2^b^	94.4 ± 3.2^b^	81.2 ± 1.3^a^
Cholesterol (mg/dl)	102.4 ± 1.2^a^	132.1 ± 2.6^c^	111.5 ± 6.2^b^	113.4 ± 0.7^b^	98.4 ± 1.1^a^
HDL-C (mg/dl)	53.1 ± 0.6^a^	49.2 ± 0.8^a^	50.2 ± 2.3^a^	51.2 ± 6.4^a^	52.4 ± 4.2^a^
LDL-C (mg/dl)	43.1 ± 2.2^a^	77.6 ± 4.2^c^	51.2 ± 1.3^b^	48.1 ± 3.1^b^	40.4 ± 2.1^a^
MDA (nmol/ml)	5.2 ± 0.1^a^	8.4 ± 2.1^d^	6.5 ± 2.4^b^	7.2 ± 0.4^c^	5.1 ± 4.1^a^
GPx (U/ml)	1435.4 ± 11.3^c^	1250.2 ± 8.8^a^	1310.6 ± 9.7^b^	1350.9 ± 10.2^b^	1421.2 ± 9.4^c^
SOD (U/ml)	29.4 ± 2.1^b^	15.2 ± 3.3^a^	27.6 ± 7.3^b^	26.4 ± 6.4^b^	28.9 ± 1.4^b^

Mean values labeled with the same superscript letter were similar (insignificance, *P* > 0.05), whereas those with different ones were significantly differed (*P* < 0.05)

### Effect of GSE and/or MSCs transplantation on liver and kidney function in different experimental groups

The highest ALT, AST, urea and creatinine levels were observed in untreated diabetic group II; these levels began to decrease upon GSE or MSCs treatment, in group III or group IV (Table 6). The best results were obtained from the combined treatment with GSE and MSCs transplantation; where no significant difference was observed in liver and kidney function parameters between healthy control group I and group V.

### Effect of GSE and/or MSCs transplantation on serum levels of lipid profile and oxidative stress in different experimental groups

No significant difference was noticed in HDL-C level among the different experimental groups. On the other hand, levels of triglycerides, cholesterol and LDL-C were highly significantly elevated in untreated diabetic group II. Group V showed comparable levels to those of control group I. Diabetes induction significantly elevated serum MDA level and reduced the antioxidant enzymes (GPx and SOD) levels. GSE administration with MSCs transplantation, in group V, diminished the elevated MDA level and increased the reduced antioxidant enzymes levels to be similar to those of control group I (Table 6).

### Effect of GSE and/or MSCs transplantation on oxidative stress, pro-inflammatory cytokines and TLR-4 in pancreatic tissue of different experimental groups

STZ administration significantly elevated pancreatic MDA level and decreased pancreatic GSH level, in comparison to control group I. This oxidative stress led to severe pancreatic inflammation that was obvious from the high levels of pro-inflammatory cytokines (IL-1β, IL-12 and TNF-α) and TLR-4. A decrease in oxidative stress and inflammation was observed in groups III and IV, treated with GSE alone or MSCs alone, in comparison to untreated diabetic group II. The combined treatment significantly returned the pancreatic tissue to its normal state, where no significant difference was observed between group V and control group I (Table 7).

**Table 7:** Effect of GSE and/or MSCs transplantation on oxidative stress and inflammation in pancreas of different experimental groups

Parameters	Healthy control group (I)	Untreated diabetic control group (II)	GSE-treated diabetic group (III)	MSCs-treated diabetic group (IV)	GSE/MSCs-treated diabetic group (V)
MDA (ng/g tissue)	23 ± 0.1^a^	80.4 ± 2.1^d^	33.6 ± 3.3^b^	46.2 ± 0.4^c^	21.1 ± 4.1^a^
GSH (pg/ g tissue)	27.9 ± 4.1^b^	18.4 ± 2.6^a^	21.4 ± 11.1^a^	19.1 ± 4.4^a^	26.4 ± 2.8^b^
IL-1β (pg/g tissue)	44.3 ± 1.6^a^	110.7 ± 0.9^c^	71.9 ± 5.4^b^	66.7 ± 0.4^b^	42.5 ± 2.8^a^
IL-12 (pg/g tissue)	56.4 ± 1.3^a^	95.5 ± 2.4^c^	61.4 ± 6.1^b^	59.7 ± 1.4^b^	53.7 ± 3.6^a^
TNF-α (pg/g tissue)	22.4 ± 0.7^a^	60.1 ± 1.6^b^	23.1 ± 1.5^a^	25.1 ± 3.6^a^	24.5 ± 2.1^a^
TLR-4 (ng/g tissue)	18.6 ± 1.4^a^	67.8 ± 4.3^c^	35.4 ± 2.2^b^	32.1 ± 0.9^b^	22.4 ± 0.4^a^

Mean values labeled with the same superscript letter were similar (insignificance, *P* > 0.05), whereas those with different ones were significantly differed (*P* < 0.05)

### Effect of GSE and/or MSCs transplantation on pancreas, liver and kidney in different experimental groups

**Fig. 3:**
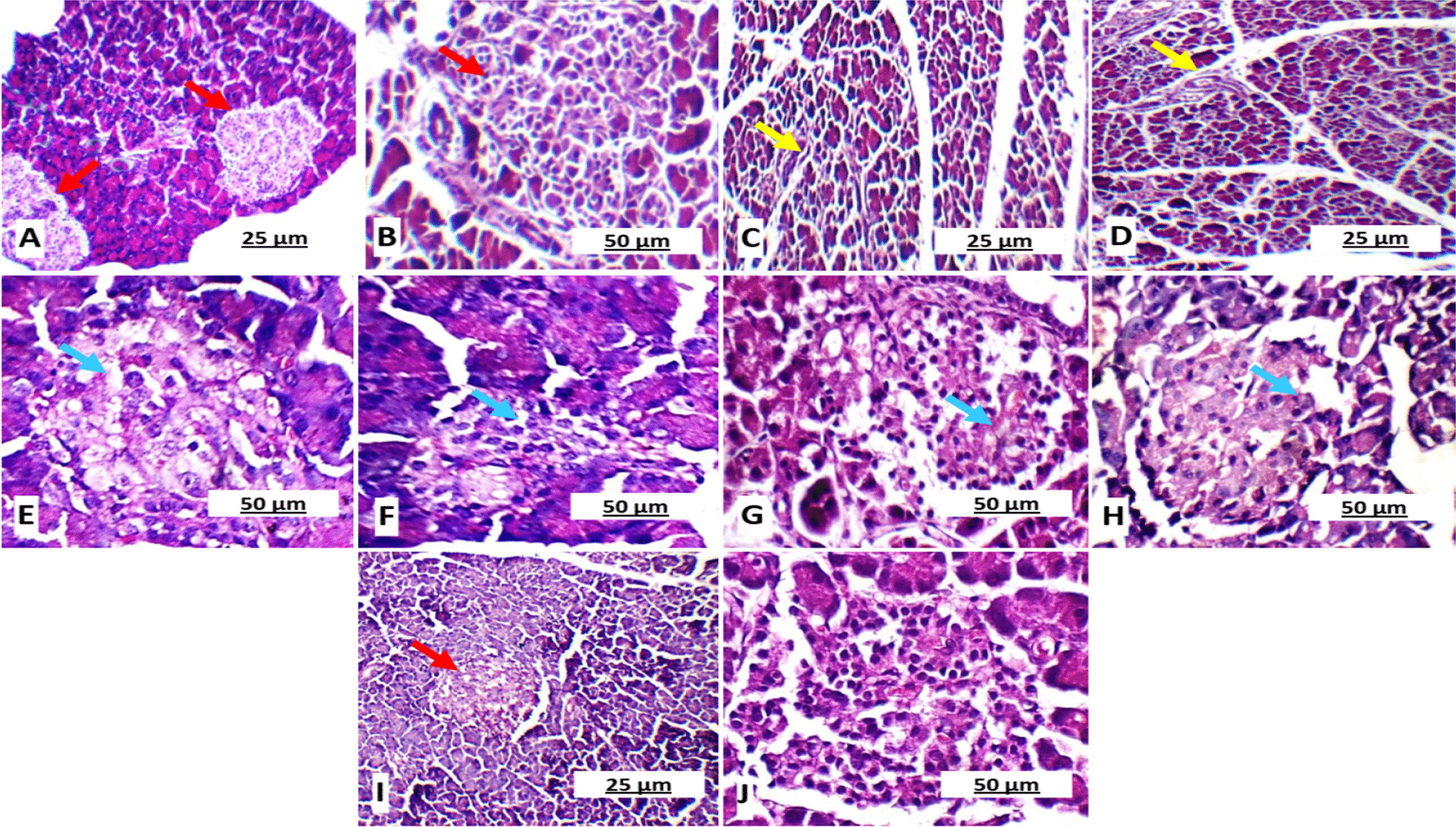
Hematoxylin and eosin stained rats’ pancreatic sections showing **A** & **B** normal pale stained islets of Langerhans (red arrow) in healthy control group I (× 100 and × 200, respectively); **C** & **D** Small-sized hypocellular islets (yellow arrow) with scattered apoptotic beta cells in untreated diabetic group II (× 200 and × 200, respectively); **E**, **F**, **G** & **H** Average-sized islets with few scattered apoptotic cells (blue arrow) in GSE-treated diabetic group III and MSCs-treated diabetic group IV (× 400); **I** & **J** normal pale stained islets of Langerhans (red arrow) in GSE/MSCs-treated diabetic group V (× 200 and × 400, respectively)

**Fig. 4:**
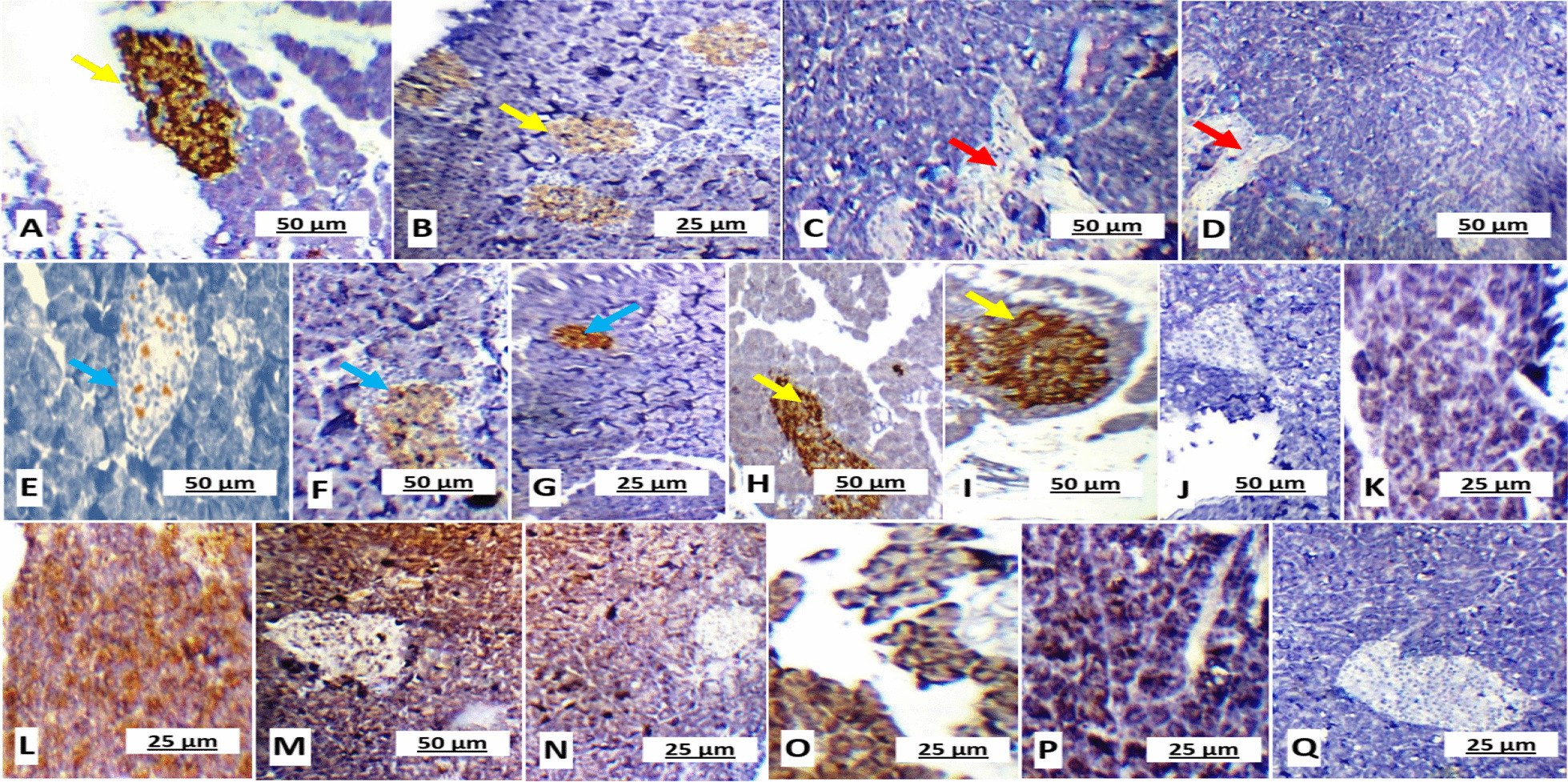
Rat pancreatic sections showing **A** & **B** marked reactivity to insulin antibodies (more than 95% of the islets) in healthy control group I (× 200); **C** &**D** Negative reactivity to insulin antibodies in untreated diabetic group II (× 200); **E** moderated reactivity to insulin antibodies in GSE-treated group III (× 400); **F** & **G** Moderated reactivity to insulin antibodies in MSCs-treated group IV (× 400 and × 100, respectively); **H** & **I** Marked reactivity to insulin antibodies (more than 95% of the islets) in GSE/MSCs-treated group V (× 200); **J** & **K** Negative reactivity to NF-κB antibodies in control group I (× 200 and × 400, respectively); **L** & **M** Marked reactivity to NF-κB antibodies in untreated diabetic group II (× 400 and × 200, respectively);); **N** Mild reactivity to NF-κB antibodies in GSE-treated group III (× 200); **O** mild reactivity to NF-κB antibodies in MSCs-treated group IV (× 400); **P** & **Q** Negative reactivity to NF-κB antibodies in GSE/MSCs-treated group V (× 400 and × 200, respectively

**Fig. 5:**
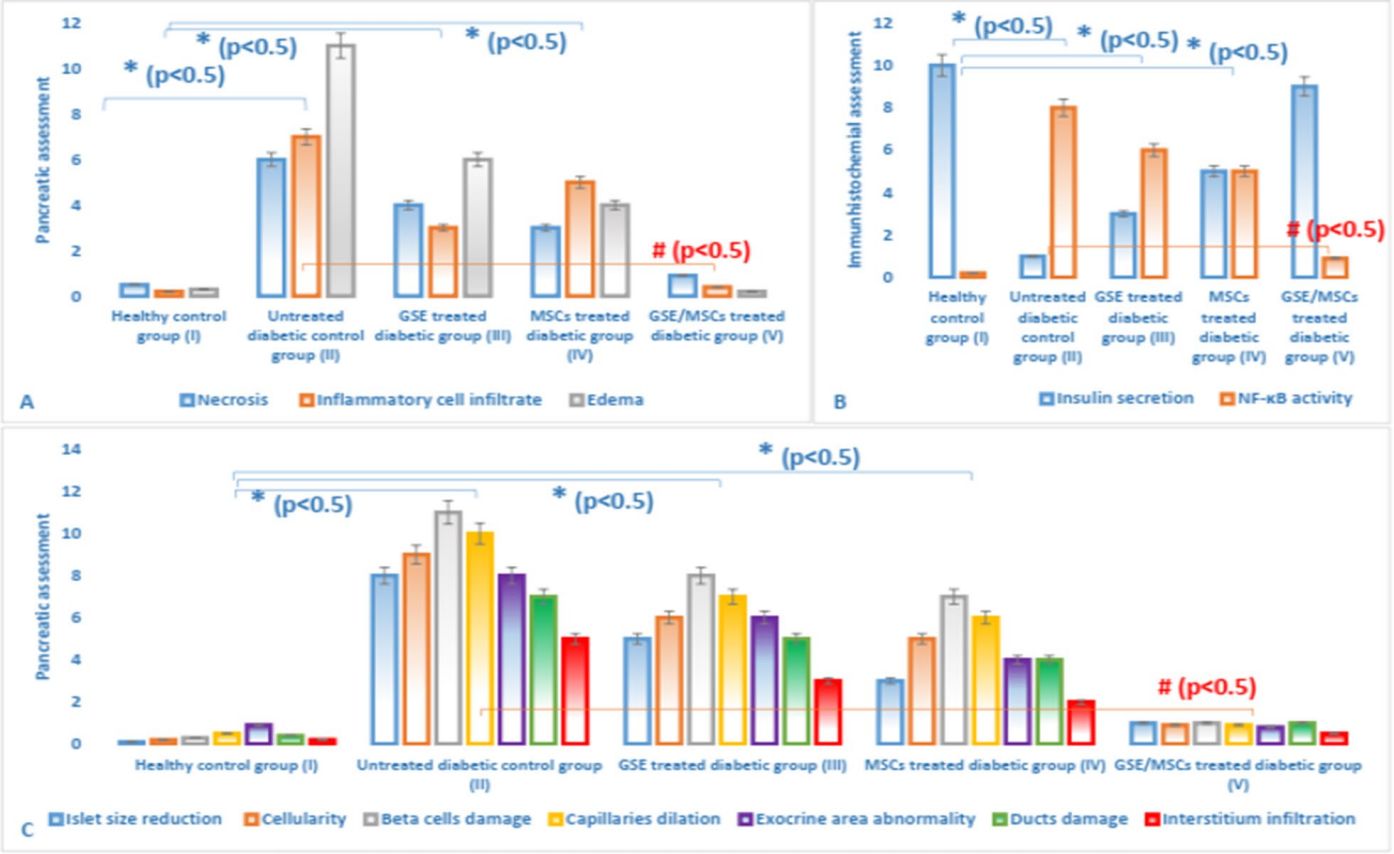
Histopathological score (**A**), immunohistochemical assessment (**B**) and morphometric analysis (**C**) of pancreatic sections in all experimental groups. Data were expressed as mean ± SD, where * indicated significance (*P* < 0.05) in comparison to healthy control group (I); and # indicated significance (*P* < 0.05) in comparison to untreated diabetic control group (II)

Pancreatic sections of control healthy group I and GSE/MSCs-treated diabetic group V showed normal pale stained islets of Langerhans (Fig. 3) with marked reactivity to insulin antibodies and negative reactivity to NF-κB antibodies (Fig. 4). On the other hand, untreated diabetic group II showed degenerated islets with marked NF-κB immunostaining and negative insulin immunostaining. GSE- or MSCs-treated groups III and IV showed average-sized islets with a few apoptotic cells and moderate insulin immunostaining. According to Fig. 5, the untreated diabetic control group II showed pancreatic sections with necrosis, edema and inflammatory cellular infiltration more than GSE- or MSCs-treated diabetic groups (*P* < 0.05). Also, the morphometric analysis and immunohistochemical assessment revealed atrophied acellular islets of Langerhans with necrotic beta cells and markedly dilated capillaries, in addition to marked NF-κB activity and diminished insulin secretion. On the other hand, co-treatment with GSE and MSCs normalized the abnormal pancreatic features that result from diabetes induction.

**Fig. 6:**
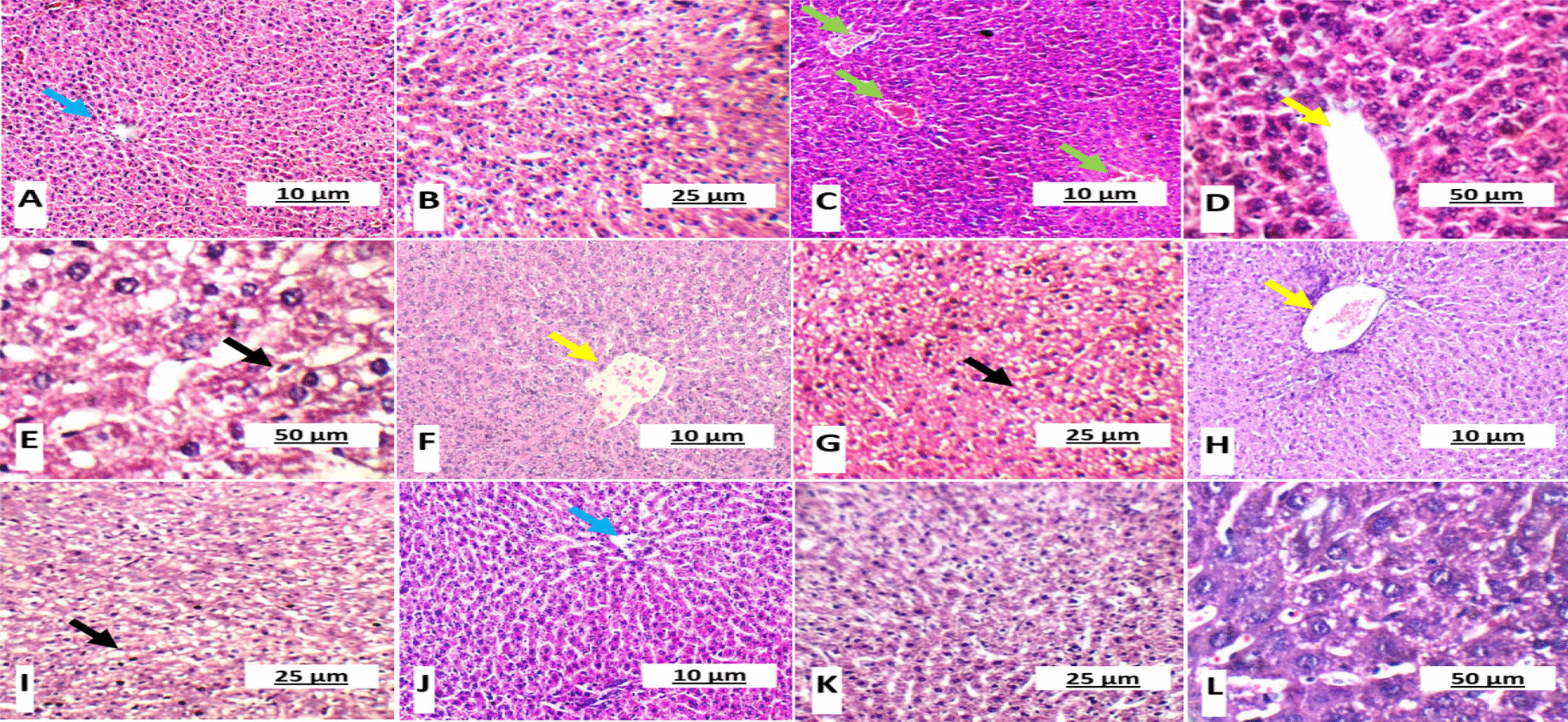
Hematoxylin and eosin stained rats’ liver sections showing **A** & **B** average central vein (blue arrow) with normal hepatocytes arranged in single cell cords in peri-portal area in healthy control group I (× 100 and × 200, respectively); **C**, **D** & **E** Marked dilated congested central vein (yellow arrow), micro- and macro-vesicular steatosis in peri-portal area (black arrow) and congested blood vessels (green arrow) in untreated diabetic group II (× 100, × 400 and × 400, respectively); **F** & **G** Marked dilated congested central vein (yellow arrow) and micro- and macro-vesicular steatosis in peri-portal area (black arrow) in GSE-treated diabetic group III (× 200); **H** & **I** marked dilated congested central vein (yellow arrow) and micro- and macro-vesicular steatosis in peri-portal area (black arrow) in MSCs-treated diabetic group IV (× 100 and × 200, respectively); **J**, **K** & **L** average central vein (blue arrow) with normal hepatocytes arranged in single cell cords in peri-portal area in GSE/MSCs-treated diabetic group V (× 100, × 200 and × 400, respectively)

**Fig. 7:**
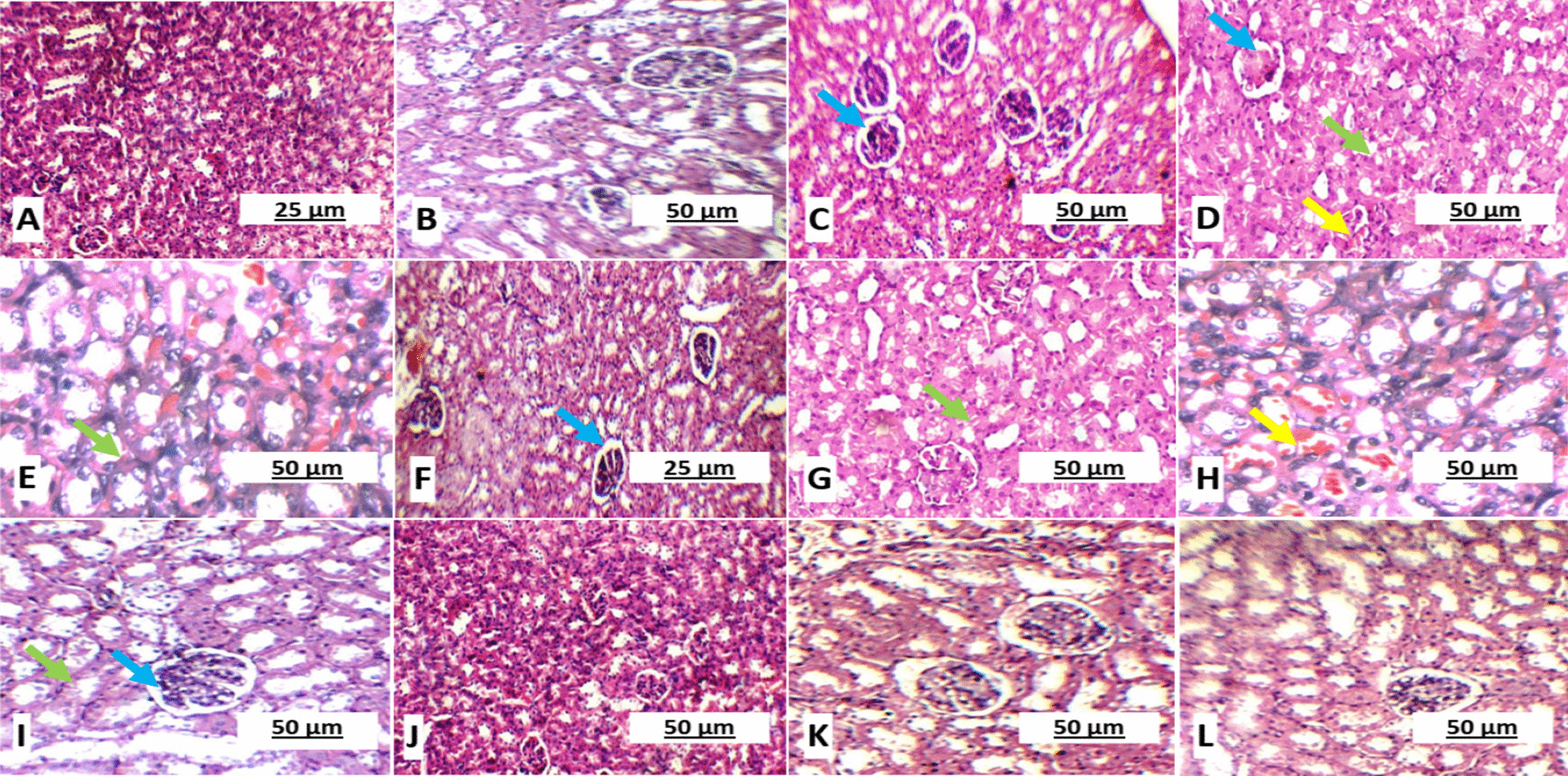
Hematoxylin and eosin stained rats’ kidney sections showing **A** & **B** average glomeruli with average renal capsule and average tubules in healthy control group I (× 100 and × 200, respectively); **C**, **D** & **E** small-sized glomerulus with widened Bowman’s space (blue arrow), proximal tubules with mildly vacuolated epithelial lining and partial loss of brush borders (green arrow) in untreated diabetic group II (× 200); **F** & **G** small-sized glomerulus with widened Bowman’s space (blue arrow), proximal tubules with mildly vacuolated epithelial lining and partial loss of brush borders (green arrow) in GSE-treated group III (× 200); **H** & **I** small-sized glomerulus with widened Bowman’s space (blue arrow), proximal tubules with mildly vacuolated epithelial lining and partial loss of brush borders (green arrow) and hemorrhage (yellow arrow) in MSCs-treated group IV (× 200); **J**, **K** & **L** average glomeruli with average renal capsule and average tubules in GSE/MSCs-treated diabetic group V (× 100, × 200 and × 200, respectively)

**Fig. 8:**
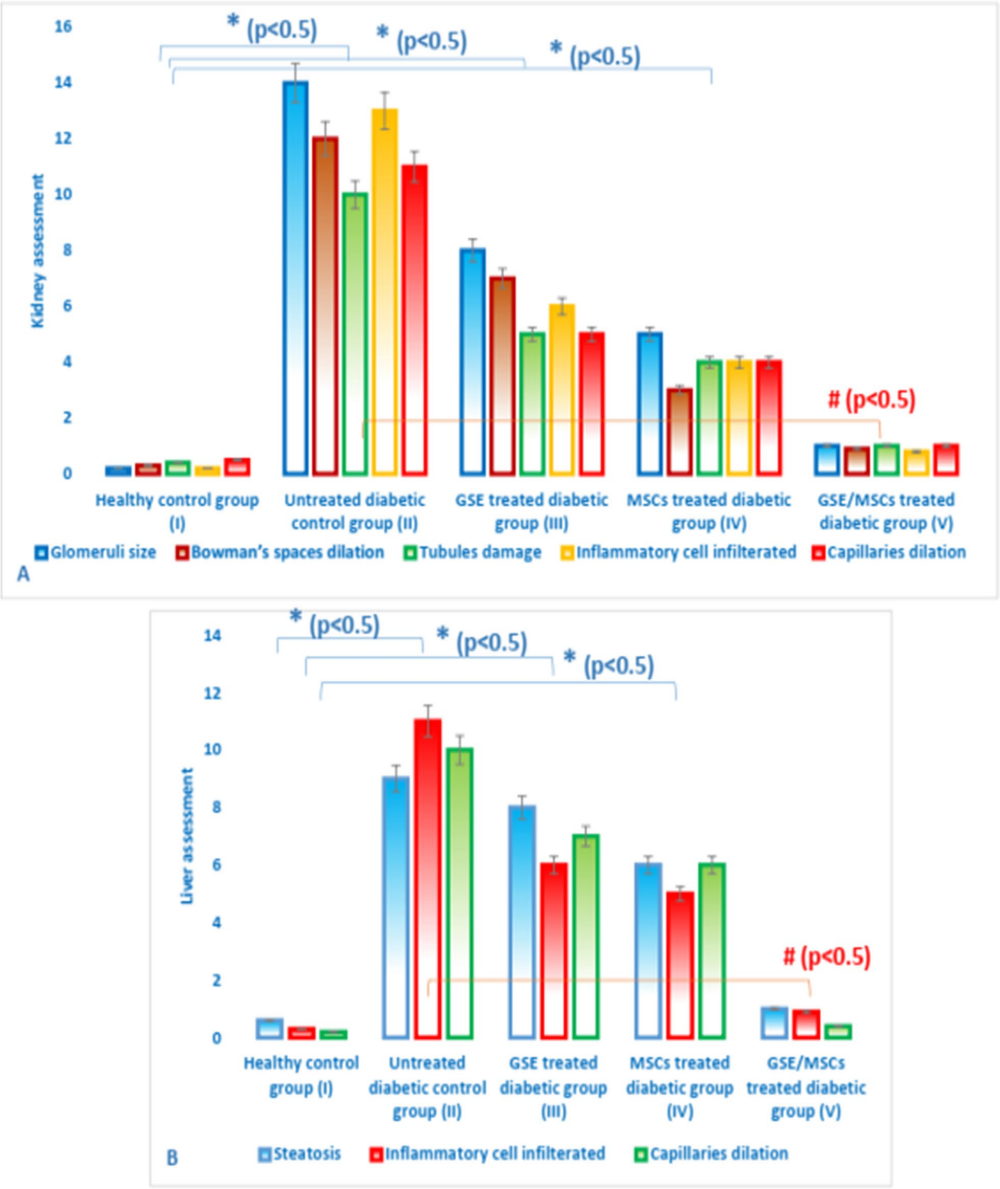
Histopathological score of kidney (**A**) and liver (**B**) in all experimental groups. Data were expressed as mean ± SD, where * indicated significance (*P* < 0.05) in comparison to healthy control group (I); and # indicated significance (*P* < 0.05) in comparison to untreated diabetic control group (II)

Normal liver sections were observed in groups I and V; where hepatocytes were arranged in single cell cords in peri-portal area (Fig. 6). Also, kidney sections of the same groups showed normal glomeruli and average tubules (Fig. 7). While, untreated diabetic gp II showed small-sized glomeruli with widened Bowman’s space, proximal tubules with mildly vacuolated epithelial lining and partial loss of brush borders (Fig. 8).

### Evaluation of GSE procyanidins in MSCs protection in vivo

**Fig. 9:**
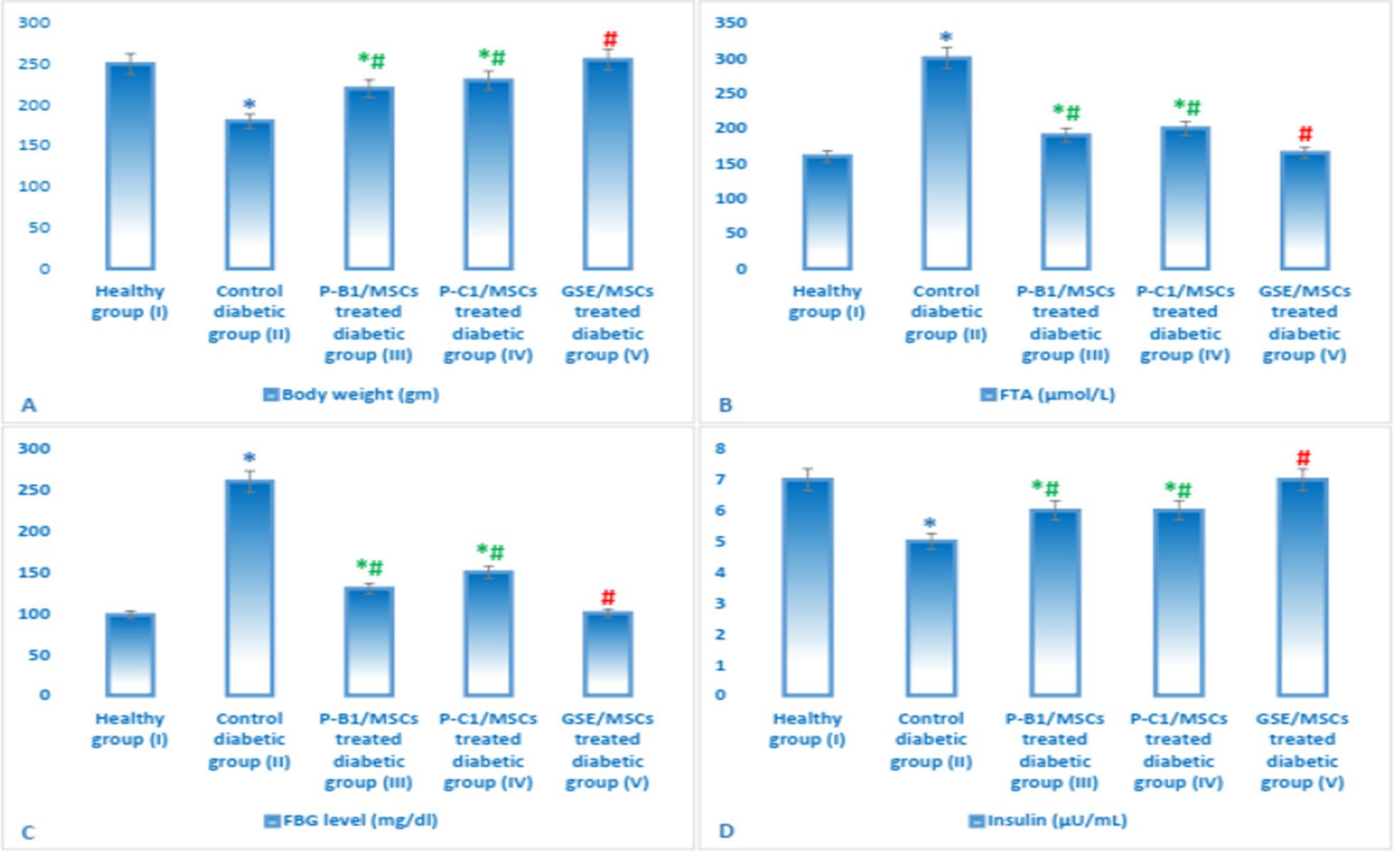
Measurements of animals’ body weight (**A**), FBG (**C**), FTA (**B**) and insulin (**D**) in different experimental groups. Data were expressed as mean ± SD, where * indicated significance (*P* < 0.05) in comparison to healthy control group (I); and # indicated significance (*P* < 0.05) in comparison to untreated diabetic control group (II)

**Fig. 10:**
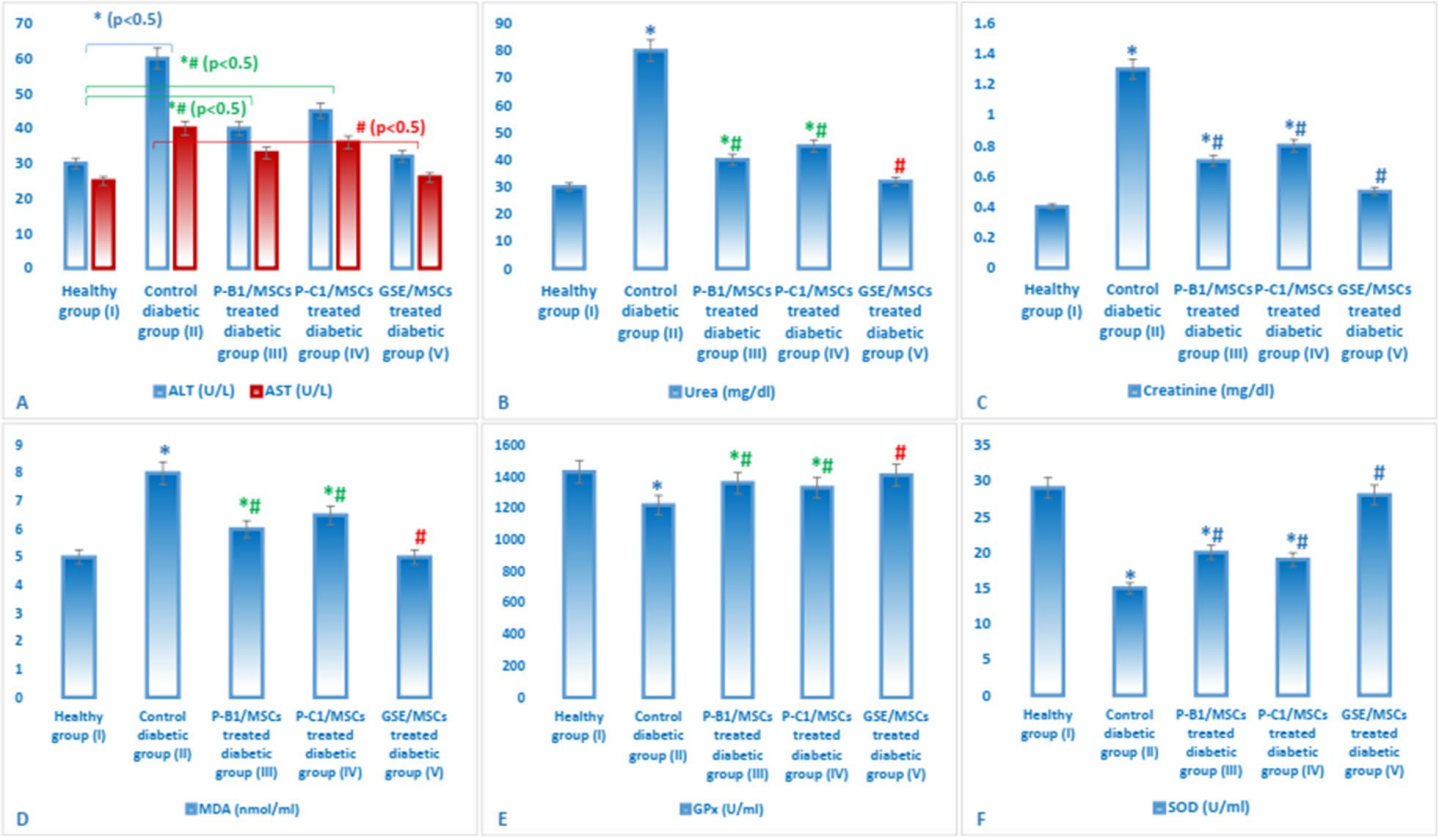
Oxidative stress [MDA (**D**), GPx (**E**) and SOD (**F**)], kidney [urea (**B**) and creatinine (**C**)] and liver function [ALT and AST (**A**)] parameters in different experimental groups. Data were expressed as mean ± SD, where * indicated significance (*P* < 0.05) in comparison to healthy control group (I); and # indicated significance (*P* < 0.05) in comparison to untreated diabetic control group (II)

Figure 9 showed that both of P-B1 and P-C1, in conjunction with MSCs transplantation, have a protective and hypoglycemic effect in diabetic rats. P-B1 and P-C1 have significantly decreased the levels of FBG, FTA, liver and kidney function parameters when compared to untreated diabetic group II. However, when comparing procyanidins-treated groups to whole GSE treated group, GSE/MSCs diabetic group V achieved more hypoglycemic and protective effects. Also, GSE/MSCs diabetic group V showed normal levels of all measured biochemical parameters (FTA, FBG, insulin, ALT, AST, urea, creatinine, MDA, SOD and GPx) when compared to procyanidins/MSCs-treated groups. Where, no significant difference was observed between group V (GSE/MSCs treated) and control group I (Figs. 9 and 10).

## Discussion

Diabetes is a progressive chronic multi-factorial disorder that arises from insulin deficiency (type I diabetes) and/or insulin resistance (type II diabetes). However, it is characterized by chronic high blood glucose levels that results from defects in the carbohydrate metabolism, leading to failure and dysfunction of many body organs such as liver, kidney and eye [38]. Several studies have investigated the role of inflammatory markers (cytokines and chemokines) in diabetes development and its complications. These researches were expected to shed light on the processes that underpin the disease's origin and progression. Increased secretion of pro-inflammatory cytokines has been linked to severe damage in pancreas of diabetic experimental rats [39]. Moreover, TLR-4 was reported to be involved in diabetes progression and insulin deficiency and/or resistance [40]. Mahmoud et al*.* [39] showed that untreated diabetic animals have a high level of TLR-4 and linked this to diabetes progression and pancreatic damage.

Due to MSCs exceptional healing abilities, they are being used in therapies for a number of ailments as a unique possible therapeutic intervention, sometimes referred to as regenerative medicine. In recent years, the therapeutic potential of MSCs for treating diabetes pathology and its repercussions has been extensively researched. Insulin-producing cells have been transplanted, paving the way for insulin-secreting pancreatic cell regeneration using stem cells [41]. MSCs have the potential to regenerate and differentiate into specialized cells such as adipocytes, chondrocytes, myocytes and osteoblasts when given the correct conditions and signals [42].

Although thousands of plants have been used both traditionally and medically, only a few plant extracts have been proven to have a specific mechanism of action on MSCs. In the presence of a specific plant extract, MSC differentiation into specific lineage-committed progenitor can help expand fields for regenerative medicine and therapy. As a result, the current study emphasizes the significance of bioactive chemicals derived from GSE in MSCs proliferation and differentiation in vivo.

Extract from different plants usually contains bioactive components like polyphenol, flavonoid, and other chemicals which are beneficial as a therapy for infectious and chronic disorders [43]. In recent years, phytochemicals generate a lot of attention due to their health benefits which necessitate more scientific studies [44]. Natural products such as vitamin D3, green tea, *Aloe vera* and GSE activate bone marrow stem cell development; also, oleic and linoleic acids activate the growth of hematopoietic stem cell [45]. MSCs proliferation and differentiation, in vitro and in vivo, were stimulated with supplementation with plant extract [46]. Plant extract has been reported to increase the proliferation of stem cells and has an anticancer effect.

Grape seeds are considered a raw material with high content of phytochemicals that can be used as a source of dietary supplements with antioxidative properties. According to Sabir et al*.* [47], the seed contains fibers (40%) and oils (10–20%). Also, the seeds contain proteins (11%), carbohydrates (26.43%), phenols (7%) and mineral salts according to Owon [48]. El-Hawary et al*.* [49] reported the presence of flavonoids, proanthocyanidins and phenolics in grape seeds. Our results showed that grape seeds had a moisture content of 9.4%, 2.6% ash, 28.2% carbohydrates, 35.5% fibers, 13.3% protein, and 10.5% lipids. Furthermore, GSE contained 11.6 mg gallic acid/g dry seed and 13.6 mg quercetin/g dry seed in terms of total phenolic compounds and flavonoids, respectively. GSE had an antioxidant activity of 89.2% and an IC50 of 34.5 g/ml.

Proanthocyanidins, oligomeric flavonoids, are a class of polyphenols found in many plants. It includes catechin and epicatechin oligomers, as well as their gallic acid esters [50]. Luca et al*.* [51] reported that grape seeds contained high content of proanthocyanidins (3532 mg/100 g). The present study used ethanol for the extraction of grape seeds due to its low toxicity according to Mandic et al*.* [19]. Kennedy et al*.* [52] showed that grape seeds can be extracted by ethanol, methanol or their mixtures. Also, Pekic et al*.* [53] reported the solubility of proanthocyanidins in ethanol and ethyl acetate and added that this method had a significant selectivity for extraction of low molecular weight flavan-3-ols. Our results showed that the average yield of GSE was 14.5 ± 1.1 g/kg dry grape seed. This yield was in agreement with Mandic et al*.* [19] who found that the yield from GSE ranged from 4.40 to 15.6 g/kg of dry seeds.

The present study used the Folin–Ciocalteou method which depended on the reducing power of the phenolic hydroxyl groups. According to this method, the total polyphenols were 11.6 ± 2.1 g/kg dry grape seed that was two times higher than the values obtained by HPLC method (5.1 ± 0.43 g/kg dry grape seed). This can be explained by the fact that Folin–Ciocalteou method detected all polyphenols with differing sensitivities; therefore, it was not highly specific. The flavan-3-ols content in Muscat of Alexandria grape seeds was determined by the vanillin assay that used ( +)-catechin as standard in calibration. Our results (11.1 g flavan-3-ols /kg dry grape seed) were in agreement with the results of Mandic et al*.* [19] who found that flavan-3-ols content in grape seeds extracted by ethanol ranged from 3.70 to 11.5 g/kg of dry seeds. Revilla et al*.* [54] and Mandic et al*.* [19] reported that the phenolic content in grape seeds was 1.84–4.07 g/kg and 3.59–11.7 g/kg, respectively.

The HPLC techniques showed that catechin (40.5%) and epicatechin (32.7%) were the predominant proanthocyanidins in Muscat of Alexandria GSE. Revilla et al*.* [54] showed that (+)-catechin (31–61%) was more predominant than (−)-epicatechin (8.6–40%) in the GSE of many examined types. And Mandic et al*.* [19] reported that ( +)-catechin and ( −)-epicatechin (38–53% and 20–36%, respectively) were the predominant phenolic compound in many tested grape seeds varieties. On the other hand, Fuleki and Ricardo da Silva [55] found that (−)-epicatechin (21–43%) was more abundant than ( +)-catechin (14–27%) in white grape seeds. Bakkalbaşi et al*.* [56], also, found that (−)-epicatechin was higher than (+)-catechin (38–64% and 36–61%, respectively).

Several studies have reported many therapeutic properties for GSE such as anticancer [57], anti-bacterial [58], antioxidant and free radicals scavenging effects [59, 60]. Balu et al. [61] showed that grape seed extract inhibited platelet aggregation and Terra et al*.* [62] reported its anti-inflammatory properties. Also, Hwang et al*.* [63] showed that grape seed extract lowered blood glucose level in diabetic rats; and Saada et al*.* [64] showed its radioprotective effect against radiation-induced damages.

In this study, administration of 300 mg/kg of GSE for 30 consecutive days with MSCs transplantation (gp V) significantly managed blood glucose levels and increased insulin secretion in experimental rats. This has been proven from the pancreatic sections examination; where islets regeneration was obvious with marked positive insulin immunostain. The anti-oxidant capability of GSE was proven from the decrease in oxidative stress biomarkers (MDA) and increase in the anti-oxidant enzymes (GSH) in pancreatic homogenates of group V rats. This was reflected on the pro-inflammatory markers (IL-1β, TNF-α, IL-12 and TLR-4) where a significant reduction in their levels was observed; and this reduction was in coincidence with negative NF-κB immunostain in group V. Also, group V showed normal lipid profile, liver and kidney functions when compared to control group I; in addition, the histopathological examination revealed normal liver and kidney architecture with no evidence of inflammation. When comparing MSCs homing between group IV (treated with MSCs only) and group V (treated with GSE and MSCs), group V showed more MSCs homing than group IV. This indicated the efficacy of GSE in reducing the oxidative stress and inflammation that results from diabetes induction and MSCs differentiation, which allowed islets of Langerhans regeneration in group V more efficiently than group IV. This astonishing power of GSE lies in its phytochemical components (Flavonoids and phenolic compounds). Moreover, when comparing the administration of procyanidins (-B1 or -C1) with the whole GSE after MSCs transplantation, we found that although P-B1 and P-C1 achieved hypoglycemic and protection efficiency on MSCs, whole GSE/MSCs was more effective. This can be attributed to the large number of bioactive components in GSE (other than procyanidins) which give GSE its power in MSCs’ protection. Further work is required to identify and test other GSE components with MSCs for ameliorating diabetes in animal model.

## Conclusion

GSE/MSCs therapy in type I-induced diabetic rats has dramatically managed homeostasis of glucose and insulin secretion, together with, improvement in the levels of inflammatory markers and oxidative stress. This is the first work to use GSE as a stimulator and protector of MSCs; the study can open a gate for treatment of diabetic patients.

## Data Availability

All data generated or analyzed during this study are included in this published article.

## Abbreviations

ALT: Alanine aminotransferase
AST: Aspartate aminotransferase
DAB: 3, 3-Diaminobenzidine
DMEM: Dulbecco’s modified Eagles medium
DPPH: 2,2-Diphenyl-1-picryl-hydrazyl-hydrate
FACS: Fluorescence-activated cell sorting
FBG: Fasting blood glucose
FTA: Fructosamine
GPx: Glutathione peroxidase
GSE: Grape seed extract
GSH: Glutathione
IC50: 50% Inhibitory concentration value
IL: Interleukin
LDL: Low-density lipoprotein
MDA: Malondialdehyde
MSC: Mesenchymal stem cell
NF-κB: Nuclear factor-kappa B
NO: Nitric oxide
P: Procyanidin
SOD: Superoxide dismutase
STZ: Streptozotocin
TLR: Toll-like receptor
TNF: Tumor necrosis factor
